# Research on Path Planning and Path Tracking Control of Autonomous Vehicles Based on Improved APF and SMC

**DOI:** 10.3390/s23187918

**Published:** 2023-09-15

**Authors:** Yong Zhang, Kangting Liu, Feng Gao, Fengkui Zhao

**Affiliations:** College of Automobile and Traffic Engineering, Nanjing Forestry University, Nanjing 210037, China; zy@njfu.edu.cn (Y.Z.); liukangting@njfu.edu.cn (K.L.); gaofeng@njfu.edu.cn (F.G.)

**Keywords:** autonomous vehicle, path planning, path tracking, artificial potential field, sliding mode control

## Abstract

Path planning and tracking control is an essential part of autonomous vehicle research. In terms of path planning, the artificial potential field (APF) algorithm has attracted much attention due to its completeness. However, it has many limitations, such as local minima, unreachable targets, and inadequate safety. This study proposes an improved APF algorithm that addresses these issues. Firstly, a repulsion field action area is designed to consider the velocity of the nearest obstacle. Secondly, a road repulsion field is introduced to ensure the safety of the vehicle while driving. Thirdly, the distance factor between the target point and the virtual sub-target point is established to facilitate smooth driving and parking. Fourthly, a velocity repulsion field is created to avoid collisions. Finally, these repulsive fields are merged to derive a new formula, which facilitates the planning of a route that aligns with the structured road. After path planning, a cubic B-spline path optimization method is proposed to optimize the path obtained using the improved APF algorithm. In terms of path tracking, an improved sliding mode controller is designed. This controller integrates lateral and heading errors, improves the sliding mode function, and enhances the accuracy of path tracking. The MATLAB platform is used to verify the effectiveness of the improved APF algorithm. The results demonstrate that it effectively plans a path that considers car kinematics, resulting in smaller and more continuous heading angles and curvatures compared with general APF planning. In a tracking control experiment conducted on the Carsim–Simulink platform, the lateral error of the vehicle is controlled within 0.06 m at both high and low speeds, and the yaw angle error is controlled within 0.3 rad. These results validate the traceability of the improved APF method proposed in this study and the high tracking accuracy of the controller.

## 1. Introduction

The automotive industry has experienced rapid development in recent years, with continuous transformation and upgrading of cars. The development of automobiles toward intelligence, electrification, networking, and sharing has greatly improved transportation and people’s daily lives [1,2,3]. The autonomous vehicle represents the most advanced technology in the industry’s development. It encompasses three main components, including environmental perception [4], path planning [5], and tracking control [6]. The first part relies on various sensors to detect the external environment and input this information into the autonomous vehicle system [7,8,9], thereby establishing the foundation for subsequent planning and control [10]. The objective of planning is to determine the most optimized path for intelligent vehicles using appropriate algorithms [11]. The function of control involves using suitable controllers to guide the vehicle along the planned path [12,13]. This study primarily focuses on the study of path planning and tracking control [14].

Path planning is one of the important aspects of intelligent vehicles. It includes two major parts, global path planning and local path planning. Global path planning is carried out throughout the entire map range, planning a rough route for vehicles from the starting point to the endpoint. The classification of common global path planning methods is as follows, graph search-based algorithms such as A* [15] and Dijkstra [16], intelligent algorithms such as the genetic algorithm [17] and particle swarm optimization [18], and machine learning-based methods such as reinforcement learning [19] and deep learning [20]. Local path planning is carried out in the environment surrounding the vehicle’s current location. It is used to plan a local collision-free path in detail.

Common local path planning algorithms include potential-based methods such as the APF method and the minimum potential energy method, rule-based methods such as the discrete method and the continuous method, and sampling-based methods such as Monte Carlo sampling [21] and inverse sampling. Most of the improved A* algorithms can effectively plan a collision-free path, as global path planning is already very mature. However, local path planning algorithms have problems such as local optima, an inability to adapt to dynamic environments, and poor security. Therefore, this study focuses on local path planning. Among them, the APF method has the advantages of simplicity, high real-time performance, and adaptability to complex environments. Therefore, the most widely used APF method was chosen for improvement.

At present, the APF method has drawbacks such as local minima, unreachable target points, and poor adaptability to traffic environments. Scholars have made a series of improvements to the APF method. Li et al. [22] proposed a minimum safe distance model for overtaking and lane-changing scenarios. The length of overtaking routes was controlled to a minimum by setting a minimum safety distance. However, only considering the minimum route ignores the dynamic constraints of the vehicle, which may result in the turning radius of the vehicle not reaching that large. Yao et al. [23] proposed a fusion method of the black hole potential field and reinforcement learning to solve the problem of local minima. Their method found target points in a multi-objective environment. At the same time, the trained autonomous vehicle quickly adapted to the scene containing new obstacles in real time. However, setting the threshold is not very easy to grasp. If the threshold is set too high, multiple gravitational fields will overlap. On the contrary, if the threshold is too low, it cannot be detected. Xie et al. [24] proposed an improved APF algorithm, which introduced the concepts of the velocity difference potential field and the acceleration difference potential field. They also proposed an optimization algorithm based on the stability of vehicles. Their experimental results demonstrated that the improved algorithm successfully enabled the safe overtaking of multiple-lane fleets. Although the generated path can meet the road constraints, dynamic constraints, and kinematic constraints in the environment set in the text, it cannot adapt to complex environments such as multiple obstacles. Duan et al. [25] proposed an improved APF method for local minima in the safe distance model. They introduced a second virtual target gravitational field. Their experiments showed that the improved APF method could effectively solve the problem of local minima. Although the safety and stability of cars were improved, they did not take into account the issues of unreachable goals and constraints of the traffic environment. Feng et al. [26] put forward a model for lateral lane changing and a model for longitudinal braking distance in order to avoid collisions. They also integrated the safety model into the APF algorithm. This allowed them to plan a collision avoidance path that satisfied stability requirements. However, their study did not consider the dynamic environment, which is too simplistic and limited in its adaptability. Yuan et al. [27] proposed lateral and longitudinal safety distance models to analyze the braking process and limit the sideslip angle. Building on these models, they improved the APF algorithm. Their simulation results demonstrated that obstacle avoidance could be achieved within a short period of time. However, they did not address the issue of unreachable targets and insufficient adaptability to different environmental conditions. These algorithms have made significant progress in enhancing planning efficiency, reducing computational costs, and complying with kinematic constraints. Nevertheless, further research and improvement are required to achieve a more efficient, accurate, and secure autonomous vehicle path planning system. Table 1 lists the methods, merits, and drawbacks of the improved APF methods mentioned in the above references.

Path tracking control is based on the planned path at the upper level and uses a specific control algorithm for tracking control [28]. Existing issues in vehicle path tracking primarily include inaccuracies in path tracking and control instability [29], as well as the presence of dynamic and kinematic constraints in the vehicle [30]. To address these concerns, researchers from both domestic and foreign backgrounds have implemented various improvement strategies for enhancing path-tracking performance. In response to the aforementioned concerns, scholars have implemented a range of measures to enhance path tracking. Chen et al. [31] proposed a hierarchical dynamic drift controller for achieving smoother tracking of general paths. The controller is divided into three layers. The first layer determines the state of the system. The second layer combines drift and typical turning control using a dynamic drift inverse model. The third layer implements a steering system and controls wheel speed. This controller can successfully achieve high tracking accuracy in real time. Wang et al. [32] improved the LQR algorithm and designed a discrete LQR controller with both feedforward and feedback components. They utilize fuzzy control methods to dynamically adjust the weight coefficients of the LQR controller. The update mechanism, based on cosine similarity, achieves the objective of reducing computational complexity. This control algorithm effectively enhances path-tracking accuracy but exhibits relatively weak steering stability. Sliding mode control(SMC), a well-established nonlinear control strategy, generates discontinuous control signals that compel the system to follow a predetermined sliding mode trajectory [33]. Nevertheless, traditional fuzzy control encounters a significant issue when the system reaches the sliding mode surface, as it leads to chattering. Hence, scholars have used diverse techniques to mitigate chattering and improve the effectiveness of the controller. Terminal sliding mode control is one such technique that suppresses chattering and reduces the convergence time in comparison with traditional SMC [34]. Ao et al. [35] introduced a super twisted sliding mode control algorithm, and with the application of backstepping techniques and experiments, they successfully demonstrated the stability and robustness of the system. Similarly, Sabiha et al. [36] developed an integral terminal SMC approach that not only guarantees finite time convergence but also enhances the convergence speed. Building upon these advancements, Wang et al. [37] proposed an adaptive integral terminal SMC method, which offers several advantages over other sliding mode controllers. While the previous enhancements demonstrated promising results, there is still room for further improvement in terms of control accuracy and stability. Consequently, this study introduces an improved sliding mode controller with error fusion based on the aforementioned SMC method to ensure accurate and stable path tracking. Table 2 shows the methods, merits, and drawbacks of the improved path-tracking methods mentioned in the above references.

**Figure 1:**
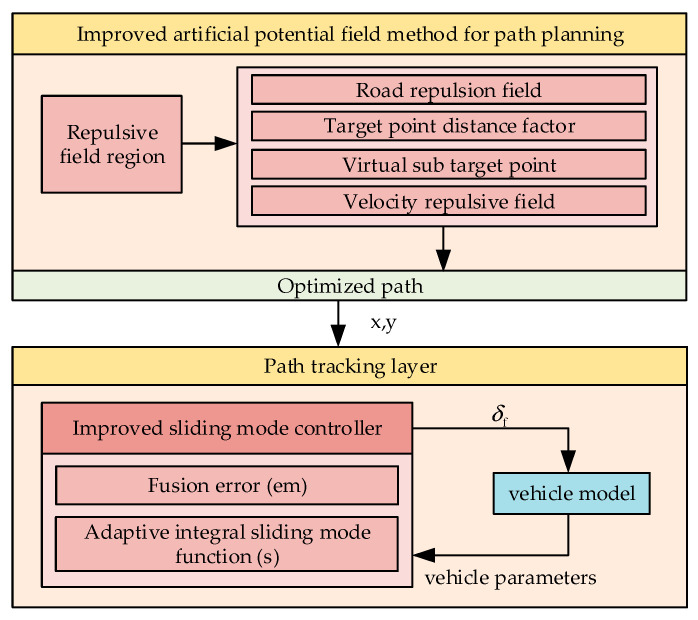
Planning and control system structure diagram.

Figure 1 is a block diagram showing the path planning and path tracking system designed in this study.

Path planning in the upper layer uses an improved APF method. Initially, the range of the obstacle repulsion field is determined. Subsequently, improvements are implemented in four aspects, including the road repulsion field, target point distance factor, virtual sub-target point, and velocity repulsion field. These enhancements lead to the generation of an improved path. The optimized path is then smoothed using a cubic Bessel curve and fed into the lower layer for path tracking. In the lower layer, an improved sliding mode controller is used for trajectory tracking. This controller incorporates fusion error and improves the sliding mode function, resulting in high-precision trajectory tracking. This validates the upper layer’s ability to generate traceable optimized paths. The main contributions of this study are as follows.

1. This study presents a method for setting the action area of a repulsive force field by analyzing the change in obstacle velocity. The detection process is divided into two areas. First, within a 120° range in front of the vehicle, a forward detection radius function is formulated based on the relative velocity between the closest obstacle and the vehicle. This function determines the detection area for obstacles in front. Similarly, a rear detection radius function is developed to cover a 240° range behind the vehicle for rear detection purposes. By considering only obstacles within the detection range, it saves computational time by ignoring irrelevant obstacle repulsion fields. This approach ensures both the accuracy and real-time performance of the path planning process.
2. In response to the problem of unreachable targets and local optima in traditional APF methods, this study introduces the concept of virtual sub-target points and a target point distance factor. These sub-target points are randomly generated within a certain radius around the target point to replace the original target point when the resultant force becomes zero during obstacle movement. By doing so, the resultant force is always maintained as non-zero, preventing the vehicle from becoming stuck in local minima. Additionally, the distance factor for the target point is included in the formula to ensure that the total force becomes zero when the vehicle reaches the target point.
3. This study presents an improved sliding mode controller that incorporates error fusion to ensure vehicle driving stability and tracking accuracy, taking into consideration both lateral and heading errors. By utilizing the improved APF algorithm, an optimized path is computed, incorporating vehicle kinematics and dynamics, which is then utilized as input for the lower controller responsible for path tracking. This aims to verify the feasibility of the planned path and evaluate the effectiveness of the enhanced tracking controller.
4. To verify the effectiveness of path planning and tracking, this study uses the Carsim–Simulink joint simulation platform. The experiment encompasses both static and dynamic scenarios. The static scene involves designing an environment with obstacles and two lanes ahead and utilizing an improved APF method to devise secure routes for vehicles. A vehicle with moving obstacles is placed in the dynamic scene, and the improved APF proposed in this study is used to generate an optimized and safe path for the autonomous vehicle. The experimental results demonstrate that the planned driving path is fully compliant with safety and road constraints, allowing for the vehicle to smoothly reach the endpoint.

## 2. Path Planning Algorithm

### 2.1. Traditional APF Algorithm

**Figure 2:**
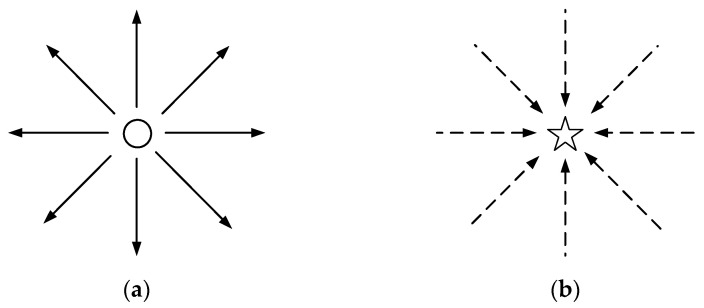
Force diagram for an autonomous vehicle in APF. (**a**) Obstacle repulsion. (**b**) Target point gravity.

The APF algorithm was initially proposed by Khatib as an algorithm for robot path planning [38]. The fundamental principle of traditional APF algorithms involves envisioning the vehicle moving within an artificially defined abstract potential field. This potential field is composed of two primary components, including a gravitational field and a repulsive field. Obstacles exert a repulsive force on the autonomous vehicle, while the target point exerts a gravitational force. Consequently, the combined forces of repulsion and gravity determine the direction of the vehicle’s movement (Figure 2). The following schematic diagram illustrates the repulsive and gravitational forces exerted by obstacles and target points on autonomous vehicles operating in APF.

Equation (1) shows the calculation for the gravitational field generated by the target point on the vehicle.

(1) $$U_{att}\left(q\right)=\frac{1}{2}k_{att}\rho^{2}(q,q_{g})$$

where $U_{att}\left(q\right)$ is the gravitational field, $k_{att}$ is the gain of the gravitational field, and $\rho (q,q_{g})$ represents the Euclidean distance between the car and the target.

The magnitude of gravity is the negative derivative of the gravitational field on the distance between the vehicle and the target point.

(2) $$F_{att}\left(q\right)=-\nabla U_{att}\left(q\right)=-k_{att}\rho (q,q_{g})=-k_{att}(q-q_{g})$$

The repulsive field function can be calculated using Equation (3).

(3) $$U_{rep}(q)=\left\{\begin{array}{c} 0,\;\rho (q,q_{g})>\rho_{0}\\ \frac{1}{2}k_{rep}[\frac{1}{\rho (q,q_{0})}-\frac{1}{\rho_{0}}],\;0\leq \rho (q,q_{g})<\rho_{0} \end{array}\right.$$

where $U_{rep}(q)$ is the size of the repulsive field, $k_{rep}$ is the gain in the repulsive field, $\rho (q,q_{g})$ represents the Euclidean distance between the car and the obstacle, and $\rho_{0}$ is the range of repulsion influence of the obstacle.

The magnitude of repulsion is the negative derivative of the repulsion field on the distance between vehicles and obstacles.

(4) $$F_{rep}(q)=-\nabla U_{rep}\left(q\right)=\left\{\begin{array}{c} 0,\;\rho (q,q_{g})>\rho_{0}\\ k_{rep}[\frac{1}{\rho (q,q_{0})}-\frac{1}{\rho_{0}}]\times \frac{1}{\rho^{2}(q,q_{0})},\;0\leq \rho (q,q_{g})<\rho_{0} \end{array}\right.$$

Based on the above analysis, in the traditional APF algorithm, the total potential field and resultant force on the intelligent vehicle are shown in Equations (5) and (6), respectively.

(5) $$U_{total}=U_{att}(q)+\sum_{i=1}^{n}U_{rep}(q)$$

(6) $$F_{total}=F_{att}(q)+\sum_{i=1}^{n}F_{rep}(q)$$

The sum of the gravitational force $F_{att}(q)$ generated by the target point and the repulsive force $F_{rep}(q)$ generated by the obstacle is the combined force $F_{total}$ on the smart car. The composition of forces controls the intelligent vehicle to avoid obstacles while driving.

### 2.2. Improved APF Method

**Figure 3:**
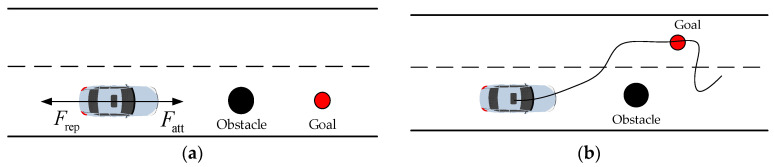
The defects of the APF method. (**a**) Local minimum. (**b**) Target unreachable.

Traditional APF suffers from the limitations of local optima and unattainable targets in the process of path planning, as depicted in Figure 3. Local optimization occurs when a vehicle halts halfway due to a zero resultant force during motion. The target becomes unreachable because the vehicle is still subjected to the repulsive force of obstacles while reaching the target point. As a result, the vehicle cannot stop at the endpoint. Additionally, the conventional APF fails to consider the constraints imposed by guardrails on both sides of the road scene, potentially leading to collisions with these barriers. Consequently, it is imperative to incorporate road constraints to ensure the safety of vehicles within a lane. Furthermore, the repulsive field function and gravitational field function of obstacles need to be enhanced for more effective performance.

**Figure 4:**
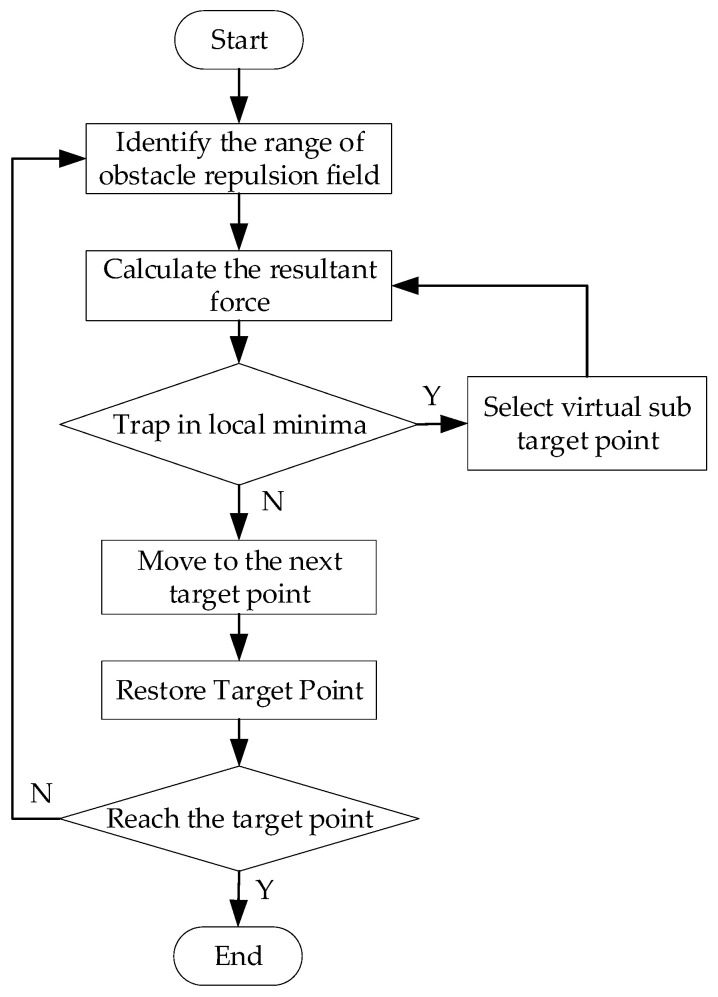
A flowchart showing the improved APF algorithm.

To address the aforementioned concerns, this study proposes an improved APF method, and Figure 4 depicts the corresponding flowchart.

Firstly, the detection area of obstacles needs to be determined, which involves delineating the scope of the repulsion field. Then, in real time, the repulsion field of obstacles within the scope of the repulsion field is calculated. Next, by considering the road repulsion field, velocity repulsion field, and gravity field, it can be determined whether the intelligent vehicle has fallen into a local minimum based on the combined force. If the vehicle has indeed fallen into a local minimum, a virtual sub-target point is selected. However, if it has not fallen into a local minimum, the vehicle moves to the next target point and the original target point is restored. If the smart car successfully reaches the target point, the cycle comes to an end. However, if the car does not reach the target point, the above operation is repeated until the target point is reached.

#### 2.2.1. The Region of Repulsive Field Action

**Figure 5:**
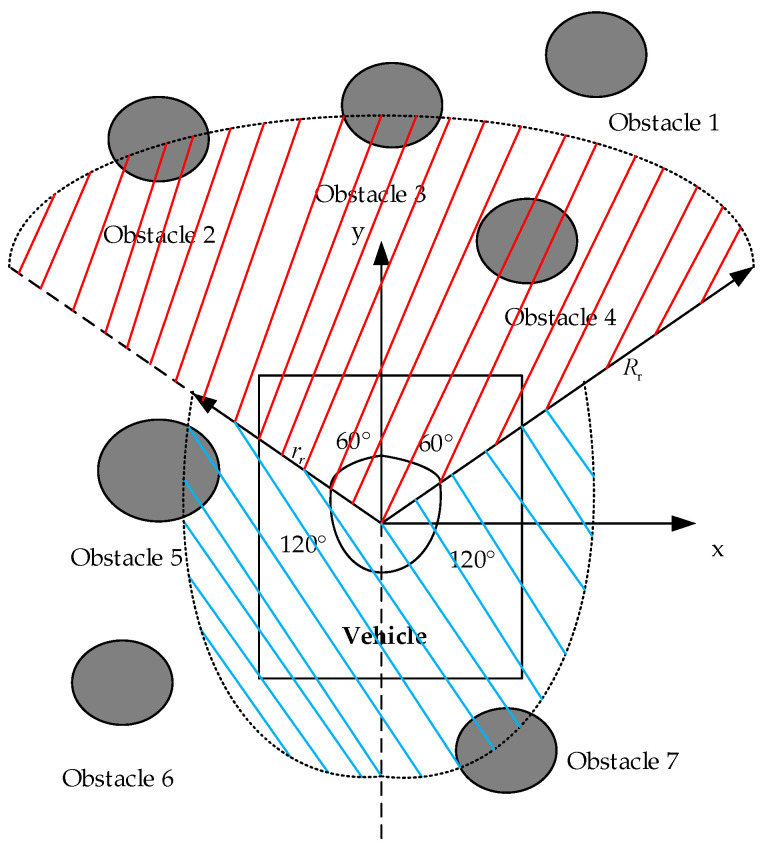
Range of obstacle repulsion field in different areas.

Considering a practical driving scenario, drivers have a field of view of 120° in front of them, with a blind spot of 240° remaining. In this study, all obstacles around the vehicle within a 360° range are taken into account. Figure 5 illustrates the range of the obstacle repulsion field.

**Figure 6:**
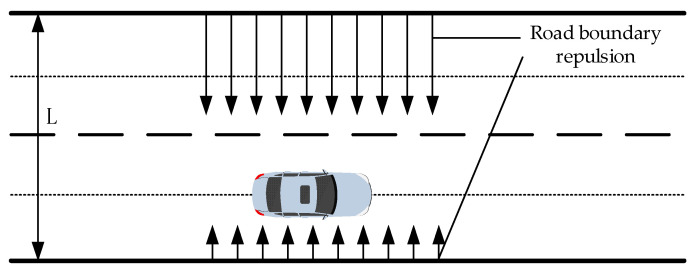
Schematic diagram showing the road repulsion field.

As depicted in Figure 5, the repulsive field operates within the shaded areas colored blue and red. The red area corresponds to the front detection zone, which is centered around the vehicle body and has a radius of $R_{r}$. This area aims to consider obstacles within a 120° arc ahead. The blue area represents the rear detection zone. It is also centered around the vehicle body, with a radius of $r_{r}$. It enables the detection of obstacles within 120° arcs on the left and right sides of the rear. While most articles focus solely on front obstacle detection, disregarding obstacles in the rear, such neglect poses a potential threat to car safety. Therefore, this study takes into consideration obstacles within the rear 240° area as well. Figure 6 illustrates that only obstacles that intersect with the blue and red shadows are taken into account. Specifically, obstacles 2, 3, 4, 5, and 7 are included in the consideration range for generating a repulsive field, while obstacles 1 and 6 are excluded. This exclusion saves time by eliminating the need to calculate the repulsive force of unrelated obstacles, ultimately achieving the target point more efficiently.

Most articles commonly use the empirical value method to select $R_{r}$ and $r_{r}$, artificially assigning a fixed value. However, this method is susceptible to human error. This study uses a real-time variable radius selection method based on obstacle velocity. The method dynamically determines $R_{r}$ and $r_{r}$ for the velocity of the front and rear obstacles, respectively. In this study, we assume that the moving obstacle is traveling in the same direction as the vehicle. 

This study selects the obstacle closest to the vehicle’s center of mass within a 120° range ahead (Obstacle 4) to determine $R_{r}$. Next, the relative velocity between this obstacle and the vehicle is calculated. $R_{r}$ can be calculated with Equation (7).

(7) $$R_{r}=\left\{\begin{array}{l} \rho (q,q_{f})+10\rho (q_{f}),\;v_{r}>10\\ \rho (q,q_{f})+v_{r}\rho (q_{f}),\;4<v_{r}\leq 10\\ \rho (q,q_{f})+4\rho (q_{f}),\;v_{r}<4 \end{array}\right.$$

where $\rho \left(q,q_{f}\right)$ represents the Euclidean distance between the car and the nearest obstacle ahead, $\rho (q_{f})$ represents the radius of the nearest obstacle ahead, and $v_{r}$ represents the relative speed between the car and the obstacle.

This study chooses the obstacle (obstacle 5) that is closest to the vehicle’s center of mass within a 240° range behind it. Then, it calculates the relative velocity between the nearest obstacle and the vehicle. This study uses Equation (8) to define the function $r_{r}$.

(8) $$r_{r}=\left\{\begin{array}{l} \rho (q,q_{r})+6\rho (q_{r}),\;v_{r}<-6\\ \rho (q,q_{r})+\left|v_{r}\right|\rho (q_{r}),\;-6<v_{r}\leq 2\\ \rho (q,q_{r})+2\rho (q_{r}),\;v_{r}>2 \end{array}\right.$$

where $\rho \left(q,q_{r}\right)$ represents the Euclidean distance between the car and the nearest obstacle behind it and $\rho (q_{r})$ represents the radius of the nearest obstacle behind it.

In summary, this study determines the front and rear detection radii by considering the relative speeds of the nearest obstacle and the vehicle within the front (0 to 120°) and rear (0 to 240°) ranges. Subsequently, the obstacle that generates the repulsive field within the defined area is identified.

#### 2.2.2. Road Repulsion Field

The traditional APF algorithm is typically used to investigate robot path planning in grid map scenes, neglecting traffic scene rules. Normally, vehicles tend to travel along the center of the road while driving, resulting in the lowest risk of colliding with guardrails. Conversely, traveling on either side increases the danger. Figure 6 illustrates a schematic diagram showing the distribution of the road repulsion field, accounting for the impact of traffic scenarios on the road within the repulsion field function. The repulsion fields on both sides of the road are set at maximum and gradually diminish toward the middle of the road.

Considering the distribution of the repulsive field in the road scenario mentioned above, it is essential to analyze the boundary repulsive field function in different segments. If the vehicle is positioned on the centerline between two sets of lanes in a relatively safe area, the repulsion field gradually weakens as its position changes. Thus, a function with a gentle trend of change is implemented. Conversely, in areas where the danger coefficient is relatively high, the repulsion field decreases rapidly as the position changes. Consequently, a function with a more pronounced trend of change is used. Considering the factors mentioned above and using two lanes as an example, the road repulsion field function is established as presented in Equation (9).

(9) $$U_{road}(q)=\left\{\begin{array}{l} k_{road1}(e^{\left|x-x_{l}\right|}-1),\;x\leq L/4\\ 2k_{road2}\left[\cos\frac{2(x-x_{l})}{L}\right],\;L/4<x<3L/4\\ k_{road3}(e^{\left|x-x_{r}\right|}-1),\;x\geq 3L/4 \end{array}\right.$$

where $k_{road1}$, $k_{road2}$, and $k_{road3}$ are the gain coefficients of the road repulsion field, *L* is the lateral width of the road, and $x_{l}$ and $x_{r}$ are the horizontal positions of the centerline of the left and right lanes, respectively.

As depicted in Figure 6, when a vehicle is positioned in the space between the centerlines of two lanes, it falls within a comparatively safe zone, resulting in a relatively minor impact from the repulsion field. Thus, a gently changing function is used for this scenario. On the contrary, when a vehicle is outside the two centerline lanes, it is in close proximity to the road boundary, indicating a high danger coefficient. Consequently, a rapidly changing function is chosen for the repulsion field in these cases. This study uses an exponential function to generate the external repulsion field. By calculating the negative derivative of the repulsive field function, the boundary repulsive force exerted by the road can be obtained, as represented in Equation (10).

(10) $$F_{road}(q)=\left\{\begin{array}{l} k_{road1}e^{\left|x-x_{l}\right|},\;x\leq L/4\\ k_{road2}\left[\sin\frac{2(x-x_{l})}{L}\right],\;L/4<x<3L/4\\ k_{road3}e^{\left|x-x_{r}\right|},\;x\geq 3L/4 \end{array}\right.$$

The vehicle should maintain its position at the center of the right lane when there are no obstacles present. In the presence of obstacles in the current lane, the vehicle should temporarily shift to the left lane, bypassing the obstacles, until it leaves the obstacle range and safely returns to the right lane. To avoid any possibility of reversing direction, it is essential to consistently drive along the centerline of the right lane.

#### 2.2.3. Target Point Distance Factor

The study introduces the concept of incorporating the distance factor of the target point to address the issue of unreachable target points. This modification ensures that the overall resultant force becomes zero when the car reaches the target point. The resulting repulsion field function is presented as follows.

(11) $$U_{rep}(q)=\left\{\begin{array}{l} \frac{1}{2}k_{rep}{\left[\frac{1}{\rho (q,q_{0})}-\frac{1}{\rho_{0}}\right]}^{2}{\left(q-q_{g}\right)}^{w},\;0\leq \rho (q,q_{0})<\rho_{0}\\ 0,\;\rho (q,q_{0})\geq \rho_{0} \end{array}\right.$$

The improved repulsion formula is obtained by performing a negative gradient operation on Equation (12).

(12) $$F_{rep}(q)=-\nabla U_{rep}(q)=\left\{\begin{array}{l} F_{rep1}+F_{rep2},\;0\leq \rho (q,q_{0})<\rho_{0}\\ 0,\;\rho (q,q_{0})\geq \rho_{0} \end{array}\right.$$

In Equation (12), $F_{rep1}$ and $F_{rep2}$ can be calculated using Equations (13) and (14).

(13) $$F_{rep1}=\left\{\begin{array}{l} k_{rep}[\frac{1}{\rho (q,q_{0})}-\frac{1}{\rho_{0}}]\times \frac{1}{\rho^{2}(q,q_{0})}{\left(q-q_{g}\right)}^{w},\;0\leq \rho (q,q_{0})<\rho_{0}\\ 0,\;\rho (q,q_{0})\geq \rho_{0} \end{array}\right.$$

(14) $$F_{rep2}=\left\{\begin{array}{l} \frac{k_{rep}}{2}{\left[\frac{1}{\rho (q,q_{0})}-\frac{1}{\rho_{0}}\right]}^{2}\times h{\left(q-q_{g}\right)}^{w-1},\;0\leq \rho (q,q_{0})<\rho_{0}\\ 0,\;\rho (q,q_{0})\geq \rho_{0} \end{array}\right.$$

The repulsion correction factor *w* (*w* > 0) significantly affects the repulsion. To prevent an unreachable target phenomenon, the value of *w* must exceed 1 to ensure that the combined repulsion at the target point is zero. Consequently, this study chooses *w* = 2 for simulation experiments.

#### 2.2.4. Virtual Sub-Target Point

**Figure 7:**
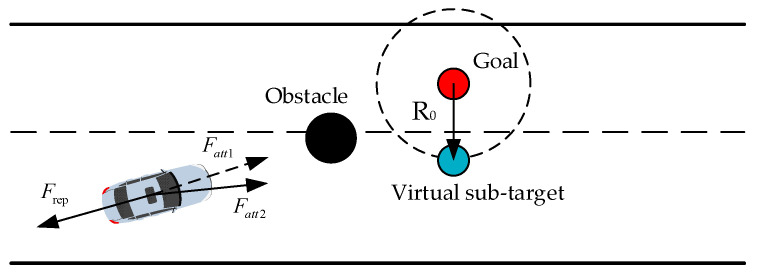
Schematic diagram showing the virtual sub-target point.

To solve the problem of reaching zero resultant force, signifying a local minimum, the concept of virtual sub-target points is introduced. A circle is formed by taking the target point as the center and $R_{0}$ as the radius, and any point on this circle is considered a virtual sub-target point. Figure 7 depicts the selection of these virtual sub-target points, where the red circle represents the actual target point and the blue circle represents the virtual sub-target point. In the case of a local minimum, the red target point is transformed into a blue virtual sub-target point. Consequently, the gravitational force exerted by the original target point on the car, denoted as $F_{att1}$, ceases to exist and is replaced with the gravitational force exerted by the virtual sub-target point on the car, designated as $F_{att2}$. Once the car escapes from the local minimum, the virtual sub-target point is removed, and the original target point is reinstated to ensure the seamless progression of car path planning.

#### 2.2.5. Velocity Repulsive Field

The preceding section solely addresses static obstacles and fails to consider the presence of moving obstacles, which may potentially lead to collisions between vehicles and moving obstacles. Therefore, we introduce a velocity repulsion field function. This function accounts for the correlation between the repulsive and gravitational fields and the square of the distance. Furthermore, as distance is directly proportional to velocity, the velocity repulsive field is also positively correlated with the square of the relative velocity. Equation (15) presents the velocity repulsive field function.

(15) $$U_{rev}(q)=\left\{\begin{array}{l} \frac{1}{2}k_{v}v_{fr}^{2},\;v_{fr}>0\cap [\alpha \in (-\frac{\pi }{3},\frac{\pi }{3})]\\ \frac{1}{2}k_{v}v_{rr}^{2},\;v_{rr}<0\cap [\alpha \in (-\frac{2\pi }{3},\frac{2\pi }{3})] \end{array}\right.$$

where $k_{v}$ is the velocity gain coefficient, $v_{fr}$ is the relative velocity between the smart car and the obstacle ahead, $v_{rr}$ is the relative velocity between the smart car and the obstacle behind, and α is the repulsive field action area mentioned earlier.

The expression for velocity repulsion can be obtained by computing the negative derivative of the velocity repulsion field.

(16) $$F_{rev}(q)=-\nabla U_{rev}(q)=\left\{\begin{array}{l} k_{v}v_{fr},\;v_{fr}>0\cap [\alpha \in (-\frac{\pi }{3},\frac{\pi }{3})]\\ k_{v}v_{rr},\;v_{rr}<0\cap [\alpha \in (-\frac{2\pi }{3},\frac{2\pi }{3})] \end{array}\right.$$

In summary, the improved resultant potential field and resultant force are calculated using Equations (17) and (18), respectively.

(17) $$U_{total}(q)=U_{att}(q)+U_{road}(q)+U_{rep}(q)+U_{rev}(q)$$

(18) $$F_{total}(q)=F_{att}(q)+F_{road}(q)+F_{rep}(q)+F_{rev}(q)$$

### 2.3. Path Optimization Algorithm for a Planned Path

In some situations, a route’s curvature is not smooth due to the discrete points. Hence, this study uses a method to simplify the route that relies on the maximum rotation constraint of the vehicle, which is performed as follows. First, start with the endpoint as the starting point for simplification, and the first two points after the endpoint as reference points for simplification. Then, check if the connecting line between the two points intersects with the obstacle and satisfies the maximum rotation constraint. If there is no intersection and the maximum rotation constraint is satisfied, then the point before the endpoint can be omitted. Repeat this process until an essential point is identified, and then use the following crucial point as the new starting point for sequential evaluation.

**Figure 8:**
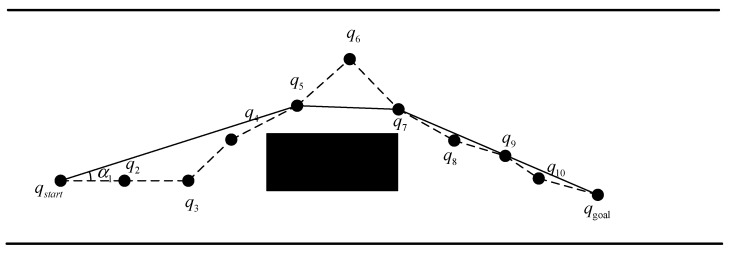
Path simplification based on the vehicle maximum rotation constraint.

As depicted in Figure 8, the schematic diagram illustrates the process of path simplification. Initially, target $q_{goal}$ is selected as the initial point for simplification and it is connected with $q_{goal}$ and $q_{9}$. If the connecting line does not intersect with any obstacles and the rotation angle falls within the specified constraint range, the nodes between $q_{goal}$ and $q_{9}$ are disregarded. Subsequently, the traversal continues with $q_{8}$, $q_{7}$, and $q_{6}$ in a sequential manner. However, if it is observed that the maximum rotation angle exceeds the constraint range upon $q_{6}$, $q_{7}$ is subsequently used as the next simplified starting point for forward traversal. Consequently, the nodes between $q_{7}$ and $q_{goal}$ are ignored. This procedure is repeated until $q_{start}$ becomes the starting point for simplification. The simplified path is represented by the solid line in Figure 8.

The simplified path mentioned above still exhibits the issue of curvature discontinuity. By utilizing a cubic B-spline curve for smoothing, a path with continuous curvature can be achieved. Assuming there are *n* + 1 control points, the k-th order B-spline curve can be defined as follows.

(19) $$P(t)=\left[p_{0},p_{1},\ldots ,p_{n}\right]\left[\begin{array}{c} B_{0,K}(t)\\ B_{1,K}(t)\\ \ldots \\ B_{n,K}(t) \end{array}\right]=\sum_{i=0}^{n}P_{i}B_{i,K}(t)$$

where P(t) represents the control point and $B_{i,K}(t)$ denotes the basis function of a cubic B-spline. Based on the de Boor–Cox formula, $B_{i,0}(t)$ and $B_{i,3}(t)$ can be inferred using Equation (20).

(20) $$\left\{\begin{array}{l} B_{i,0}(t)\left\{\begin{array}{l} 1,\;t_{i}\leq t\leq t_{i+1}\\ 0,\;Otherwise \end{array}\right.\;K=1\\ B_{i,3}(t)=\frac{\left(t-t_{i})B_{i,2}(t\right)}{t_{i+3}-t_{i}}+\frac{\left(t_{i+4}-t)B_{i+1,2}(t\right)}{t_{i+4}-t_{i+1}},\;K\geq 2 \end{array}\right.$$

In this study, a cubic uniform B-spline curve is selected to smooth the planned path, and the repeatability of the nodes is set to 3 at both ends. The basis function can be expressed using Equation (21).

(21) $$\left\{\begin{array}{l} B_{0,3}(t)=\frac{1}{6}{\left(1-t\right)}^{3}\\ B_{1,3}(t)=\frac{1}{6}\left(3t^{3}-6t^{2}+4\right)\\ B_{2,3}(t)=\frac{1}{6}\left(-3t^{3}+3t^{2}++3t+1\right)\\ B_{3,3}(t)=\frac{1}{6}t^{3} \end{array}\right.$$

The parameter node’s vector interval is defined as [0, 1]. By incorporating Equation (21) into Equation (19), we can derive the expression for the cubic quasi-uniform B-spline curve as shown below.

(22) $$P(t)=P_{0}B_{0,3}(t)+P_{1}B_{1,3}(t)+P_{2}B_{2,3}(t)+P_{3}B_{3,3}(t),\;t\in \left[0,1\right]$$

## 3. Path Tracking Controller

### 3.1. Vehicle Model

**Figure 9:**
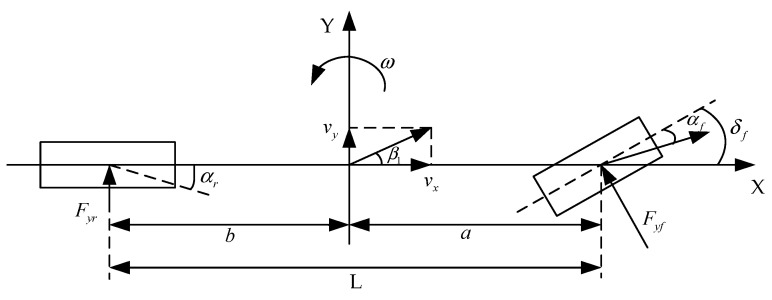
Two-degree-of-freedom vehicle model.

The objective of this study is to accomplish tracking control of a planned path. The dynamic model used in this study is a typical two-degree-of-freedom model, as depicted in Figure 9.

The model is used to perform dynamic analysis on the lateral and longitudinal directions of the model to derive the dynamic equation of the vehicle with two degrees of freedom.

(23) $$\left\{\begin{array}{l} \overset{\cdot }{\beta }=\frac{\left(k_{1}+k_{2}\right)}{mv_{x}}\beta +\left(\frac{ak_{1}-bk_{2}}{mv_{x}^{2}}-1\right)\omega -\frac{k_{1}}{mv_{x}}\delta_{f}\\ \overset{\cdot }{\omega }=\frac{ak_{1}-bk_{2}}{I_{z}}\beta +\frac{a^{2}k_{1}+b^{2}k_{2}}{I_{z}v_{x}}\omega -\frac{ak_{1}}{I_{z}}\delta_{f} \end{array}\right.$$

In Equation (23), β and ω respectively represent the side slip angle and yaw rate of the vehicle’s center of mass. The symbols $k_{1}$ and $k_{2}$ represent the side slip stiffness of the front and rear wheels, respectively, while represents the mass of the vehicle. The distances from the center of mass of the vehicle to the front and rear axles are represented by a and b. The longitudinal and lateral velocities of the vehicle are denoted as $v_{x}$ and $v_{y}$, respectively. Additionally, $\delta_{f}$ represents the front wheel angle of the car, and $I_{z}$ represents the car’s rotational inertia around the z-axis.

**Figure 10:**
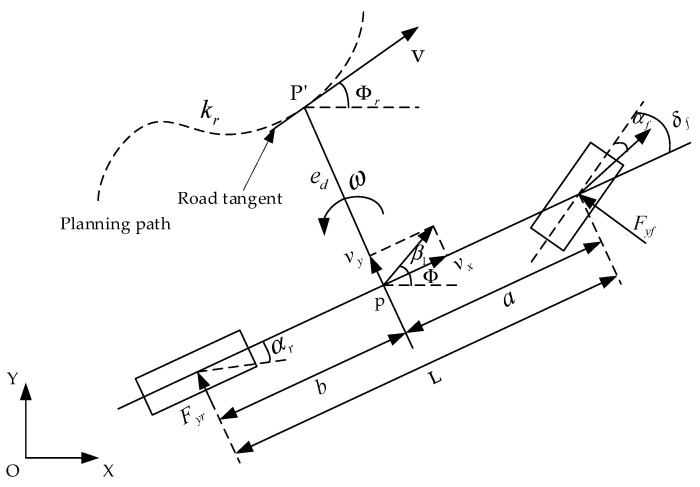
Vehicle tracking error model.

The vehicle tracking model serves as the foundation for designing the trajectory-tracking controller. The trajectory-tracking controller enhances the accuracy of vehicle trajectory tracking by dynamically adjusting the front wheel angle based on the car’s lateral and heading errors in real time. Figure 10 illustrates the vehicle tracking error model. In Figure 10, p represents the centroid of the car, p’ represents the projection point of the centroid toward the center of the road, indicates the curvature of the road, and $e_{d}$ indicates the lateral error.

The position of the car in the vehicle coordinate system is denoted as (*x*, *y*), while its position in the geodetic coordinate system is denoted as (*X*, *Y*). The origin of the vehicle coordinate system, representing the vehicle center of mass, is denoted as (*X*_0_, *Y*_0_). Additionally, the linear velocity of the vehicle is represented by *v*, and the heading angle of the vehicle is denoted as ϕ. Consequently, the position of the vehicle in the geodetic coordinate system is expressed as Equation (24).

(24) $$\left\{\begin{array}{l} X=X_{0}+\int_{0}^{t}v\cos\phi d_{t}\\ Y=Y_{0}+\int_{0}^{t}v\sin\phi d_{t} \end{array}\right.$$

The heading error ${∆}_{\phi }$ is calculated by subtracting the reference value $\phi_{r}$ from the heading angle ϕ, as shown in Equation (25).

(25) $${∆}_{\phi }=\phi -\phi_{r}$$

The lateral error is calculated in using Equation (26).

(26) $$\overset{\cdot }{e_{d}}=v_{x}\sin{∆}_{\phi }+v_{y}\cos{∆}_{\phi }$$

Considering that the lateral error is negligible, Equation (26) can be equivalently represented as Equation (27).

(27) $$\overset{\cdot }{e_{d}}=v_{x}{∆}_{\phi }+v_{y}$$

The reference trajectory yaw rate is calculated using Equation (28).

(28) $$\omega_{r}=\overset{\cdot }{\phi_{r}}=\frac{v_{x}}{R}=v_{x}\cdot k_{r}$$

where *R* is the turning radius.

Equation (29) can be obtained by taking the derivative of the heading error.

(29) $$\overset{\cdot }{{∆}_{\phi }}=\omega -\omega_{r}=\omega -v_{x}\cdot k$$

The derivative of the reciprocal of both the lateral error and heading error can be computed simultaneously.

(30) $$\left\{\begin{array}{l} \overset{\cdot \cdot }{e_{d}}=\overset{\cdot }{v_{x}}{∆}_{\phi }+v_{x}\overset{\cdot }{{∆}_{\phi }}+\overset{\cdot }{v_{y}}\\ \overset{\cdot \cdot }{{∆}_{\phi }}=\overset{\cdot }{\omega }-\overset{\cdot }{v_{x}}k_{r}-v_{x}\overset{\cdot }{k_{r}} \end{array}\right.$$

Equations (23) and (30) can be combined to obtain Equation (31).

(31) $$\left\{\begin{array}{l} \overset{\cdot \cdot }{e_{d}}=\frac{k_{1}+k_{2}}{mv_{x}}\overset{\cdot }{e_{d}}-\frac{k_{1}+k_{2}}{m}{∆}_{\phi }+\frac{ak_{1}+bk_{2}}{mv_{x}}\overset{\cdot }{{∆}_{\phi }}-\frac{ak_{1}}{m}\delta_{f}+\frac{ak_{1}-bk_{2}}{m}k_{r}-k_{r}v_{x}^{2}+\overset{\cdot }{v_{x}}{∆}_{\phi }\\ \overset{\cdot \cdot }{{∆}_{\phi }}=\frac{ak_{1}-bk_{2}}{I_{z}v_{x}}e_{d}-\frac{ak_{1}-bk_{2}}{I_{z}}{∆}_{\phi }+\frac{a^{2}k_{1}+b^{2}k_{2}}{I_{z}v_{x}}\overset{\cdot }{{∆}_{\phi }}-\frac{ak_{1}}{I_{z}}\delta_{f}+\frac{a^{2}k_{1}+b^{2}k_{2}}{I_{z}}k_{r}-\overset{\cdot }{v_{x}}k_{r}-v_{x}\overset{\cdot }{k_{r}} \end{array}\right.$$

The state variables of the system are defined as $x_{1}=e_{d}$, $x_{2}=\overset{\cdot }{e_{d}}$, $x_{3}={∆}_{\phi }$, $x_{4}=\overset{\cdot }{{∆}_{\phi }}$, and the input variable as $u=\delta_{f}$. $\delta_{f}$ represents the front wheel angle of the vehicle.

### 3.2. Design of an Improved SMC Controller

The path-tracking method for controlling the general path is based on the analysis of lateral error and heading error. Additionally, a third control method utilizes the fusion of lateral error and heading error for control. In this study, we use the comprehensive approach of considering both lateral and heading errors in trajectory tracking. The designed fusion error function is presented in Equation (32).

(32) $$e_{m}=x_{m1}e_{d}+x_{m2}{∆}_{\phi }$$

where $x_{m1}$ represents the lateral error coefficient and $x_{m2}$ represents the heading error coefficient.

From the expression, it is evident that the focus of the error can be adjusted by setting the two weight values, $x_{m1}$ and $x_{m2}$, allowing for more accurate control over either the heading angle or lateral deviation. Given the simplified working conditions in this study, with low vehicle and obstacle speeds in the simulation, stability control is straightforward. After careful calibration, it is determined that the optimal tracking effect is achieved by setting $x_{m1}$ and $x_{m2}$ to 3 and 0.1, respectively. The design of the sliding mode function for the SMC controller, utilizing the fusion error $e_{m}$ mentioned earlier, is expressed in Equation (33).

(33) $$s=\lambda_{1}e_{m}+\lambda_{2}\overset{\cdot }{e_{m}}+\lambda_{3}\int_{0}^{t}e_{m}d_{t}$$

where $\lambda_{1}$, $\lambda_{2}$, and $\lambda_{3}$ are sliding mode coefficients.

The derivative of Equation (34) is taken, resulting in the following.

(34) $$\overset{\cdot }{s}=\lambda_{1}\overset{\cdot }{e_{m}}+\lambda_{2}\overset{\cdot \cdot }{e_{m}}+\lambda_{3}e_{m}$$

The drawback of SMC is the generation of chattering. Thus, to enhance the system’s stability, a convergence rate is used. The commonly used convergence rate, aiming to improve system stability, is selected.

(35) $$\overset{\cdot }{s}=-\varepsilon_{1}\mathrm{sgn}(s)-\varepsilon_{2}s$$

where $\varepsilon_{1}$ and $\varepsilon_{2}$ are the general convergence rate coefficients.

To further reduce system chattering, the hyperbolic tangent function tanh(s) replaces the sign function sgn(*s*). Furthermore, Equations (34) and (35) are amalgamated to obtain the following expression.

(36) $$\left\{\begin{array}{l} \overset{\cdot }{e_{m}}=\omega_{1}+\omega_{2}+\omega_{3}\delta_{f}\\ \begin{array}{l} \omega_{1}=x_{m1}(\frac{k_{1}+k_{2}}{mv_{x}}\overset{\cdot }{e}-\frac{k_{1}+k_{2}}{m}{∆}_{\phi }+\frac{ak_{1}-bk_{2}}{mv_{x}}\overset{\cdot }{{∆}_{\phi }})+\\ x_{m2}(\frac{ak_{1}-bk_{2}}{mv_{x}}e-\frac{ak_{1}-bk_{2}}{I_{z}}{∆}_{\phi }+\frac{a^{2}k_{1}+b^{2}k_{2}}{I_{z}v_{x}}{∆}_{\phi }) \end{array}\\ \begin{array}{l} \omega_{2}=x_{m1}(\frac{ak_{1}-bk_{2}}{m}R-Rv_{x}^{2}+\overset{\cdot }{v_{x}}{∆}_{\phi })+\\ x_{m2}(\frac{a^{2}k_{1}+b^{2}k_{2}}{I_{z}}R-\overset{\cdot }{v_{x}}R-v_{x}\overset{\cdot }{R}) \end{array}\\ \omega_{3}=-\frac{ak_{1}}{m}x_{m1}-\frac{ak_{1}}{I_{z}}x_{m2} \end{array}\right.$$

(37) $$\delta_{f}=u=-\frac{1}{\lambda_{2}\omega_{3}}(\lambda_{2}\omega_{1}+\lambda_{2}\omega_{2}+\lambda_{1}\overset{\cdot }{e_{m}}+\lambda_{3}e_{m}+\varepsilon_{1}\tanh(s)+\varepsilon_{2}s)$$

## 4. Simulation Analysis

### 4.1. Simulation Analysis of Path Planning

This study investigates simulations in both static and dynamic states, where the static scenarios are further divided into lane changing and overtaking. It compares parameters such as length, time, and curvature of the path planned using three methods, including the general APF (G-APF), improved APF (I-APF), and optimized and improved APF (O-I-APF). The simulation results confirm the advantages of the improved APF approach, namely, its fast convergence speed, short planning time, and high safety in path planning. The simulation environment is a two-dimensional space, with map sizes of 65 m × 8 m and 60 m × 8 m. Each road has a width of 3.5 m.

(1) Scenario 1: Changing lanes (static)

**Figure 11:**
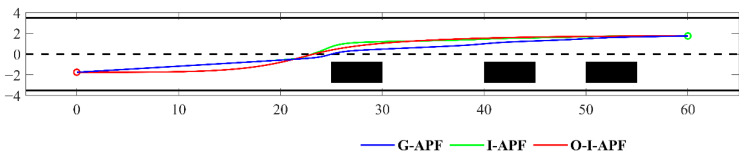
Comparison of planning paths in scenario one.

Scenario 1 presents a static lane change situation on a road measuring 65 m × 8 m. The obstacle ahead of the car has a constant speed of zero, starting from coordinates (0, −1.75) and concluding at (60, 1.75). In Figure 11, we can observe three stationary black rectangles serving as obstacles, each measuring 3.5 m × 1.8 m. The blue solid line represents the path planned using the G-APF method, while the green solid line represents the path planned using the I-APF method proposed in this study. Lastly, the red solid line represents the path planned using the O-I-APF method proposed in this study.

From Figure 11, it is evident that the initial path, prior to improvement, exhibits poor safety. During the initial obstacle avoidance process, the distance from the obstacle is insufficiently large, thus posing a risk of collision. In contrast, the improved path planning incorporates a sufficient safety distance from the obstacle, thereby enhancing the safety of the planned path. The path of the G-APF method fails to promptly align with the center of the road, which is not in accordance with traffic regulations. Conversely, the I-APF method incorporates road constraints and ensures alignment with the center of the road during non-obstacle avoidance processes, reflecting the actual driving habits of drivers. Table 3 lists the simulation data for the aforementioned path planning.

From Table 3, it can be seen that the planning time of I-APF is shortened by 0.013 s compared with G-APF. The maximum curvature of I-APF is 0.201, which is 74.49% shorter than that of G-APF. Although the path length of I-APF is not shortened, I-APF is optimized to obtain O-I-APF. Its path length is shortened by 0.14 m compared with G-APF. Its maximum curvature becomes 0.033, which is 16.42% of I-APF.

**Figure 12:**
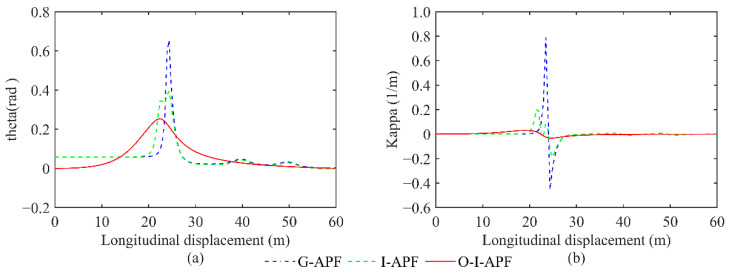
Comparison between the heading angle and curvature calculated using different algorithms. (**a**) Changes in the front wheel angle. (**b**) Changes in route curvature.

Figure 12 displays a comparison between the heading angle and curvature for the three different methods, including G-APF, I-APF, and O-I-APF. Specifically, the G-APF method is represented with the blue dashed line, the I-APF method is represented with the green dashed line, and the O-I-APF is represented with the red solid line.

Figure 12 illustrates that the O-I-APF method demonstrates a smoother transition in the turning angle of the path and curvature change in the planned route, wherein the maximum curvature is regulated within 0.04, ultimately leading to enhanced outcomes.

(2) Scenario 2: Static overtaking

**Figure 13:**
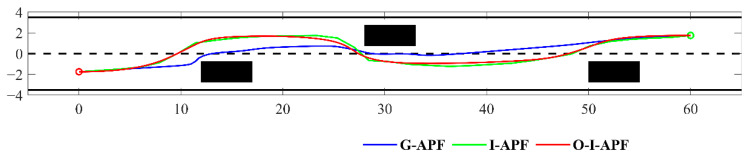
Comparison of planning paths in scenario two.

Scenario 2 pertains to a static overtaking situation on a 65 m × 8 m road. The speed of the obstacle in front of the car is set to zero, moving from position (0, −1.75) to (60, 1.75). Figure 13 illustrates three black rectangles representing stationary obstacles with dimensions of 3.5 m × 1.8 m. The blue solid line represents the path planned using the G-APF method, the green solid line represents the path planned using the I-APF method proposed in this study, and the red solid line represents the path planned using the O-I-APF method proposed in this study.

Figure 13 illustrates that the original planned path before improvement had poor safety. During the path planning process, it was in close proximity to the top left corner of the first obstacle and the bottom left corner of the second obstacle, posing a collision risk. Conversely, the I-APF method resulted in a path with a safe distance from all three obstacles, leading to a higher level of safety for the planned route. Additionally, the G-APF regulations forced the path to strictly follow the centerline of the road, disregarding common driving habits and traffic rules. In contrast, the I-APF regulations effectively guide the path along the middle of the road, aligning with conventional driving requirements. Table 4 shows the simulation data for the aforementioned path planning.

From Table 4, it can be seen that the planning time of I-APF is 0.01 s shorter than that of G-APF. The maximum curvature of I-APF is 0.797, which is 31.53% shorter than that of G-APF. Although the path length of I-APF is not shortened, I-APF is optimized to obtain O-I-APF. Its path length increases by 0.47 m compared with G-APF, but the small increment can be ignored. Its maximum curvature becomes 0.236, which is 29.61% of I-APF.

**Figure 14:**
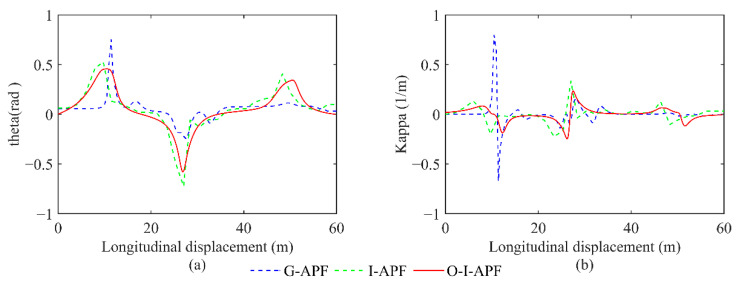
Comparison between the heading angle and curvature calculated using different algorithms. (**a**) Changes in the front wheel angle. (**b**) Changes in route curvature.

Figure 14 displays a comparison between the heading angle and curvature for the three different methods.

From Figure 14, it is apparent that the O-I-APF method exhibits a more gradual variation in the turning angle of the path. The curvature change in the planned route is also stable, displaying a smaller curvature. Its maximum curvature is constrained to 0.24, leading to superior outcomes.

(3) Scenario 3: Dynamic overtaking

**Figure 15:**
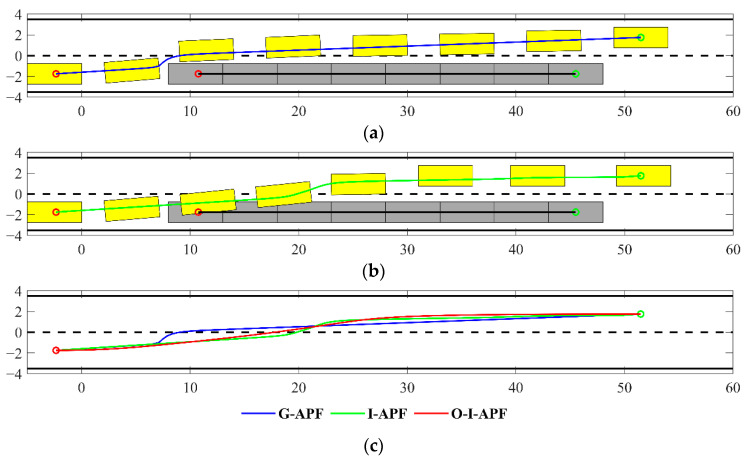
Comparison between the planning paths in scenario 3. (**a**) Path planning before improvement. (**b**) Improved path planning. (**c**) Comparison among planning paths.

Scenario 3 involves an overtaking situation on a road measuring 63.5 m × 8 m. The obstacle in front of the autonomous vehicle has a speed. The starting and ending points of autonomous vehicle are respectively at (−2.35, −1.75) and (53.1.75). Figure 15 illustrates a gray rectangle representing an obstacle vehicle with a fixed speed of 5 m/s, while the yellow rectangle represents an autonomous vehicle traveling at a speed of 8 m/s. Both vehicles have uniform dimensions of 4.7 m × 1.8 m. In Figure 15, (a) represents the real-time path planned using the G-APF method; (b) depicts the path planned using the I-APF method discussed in this study; and lastly, (c) illustrates the composite representation of the paths planned using all three algorithms on a single graph. The blue line displays the path planned using the G-APF method, the green line represents the path planned using the I-APF method, and the red line shows the path planned using the O-I-APF method.

From Figure 15, it is evident that the G-APF method promptly avoids obstacles at the outset. However, at this juncture, the obstacle in front of the vehicle is shifted from its original position. This results in premature and rapid obstacle avoidance, which lacks a required reaction time and compromises the smoothness of the path. The I-APF method generates a path that overlaps with the obstacle’s previous position upon second arrival, ensuring there is no collision. Moreover, the obstacle avoidance action exhibits a slight delay, enabling the driver to seamlessly transition to another lane with sufficient reaction time. Table 5 lists the simulation data for the aforementioned path planning.

From Table 5, it can be seen that the path length of I-APF is shortened by 0.104 m compared with G-APF. I-APF reduces the planning time by 0.005 s compared with G-APF. The maximum curvature of I-APF is 0.167, which is 87.82% shorter than that of G-APF. The path length of O-I-APF is shortened by 0.383 m compared with I-APF. Its maximum curvature becomes 0.014, which is 8.38% of I-APF.

**Figure 16:**
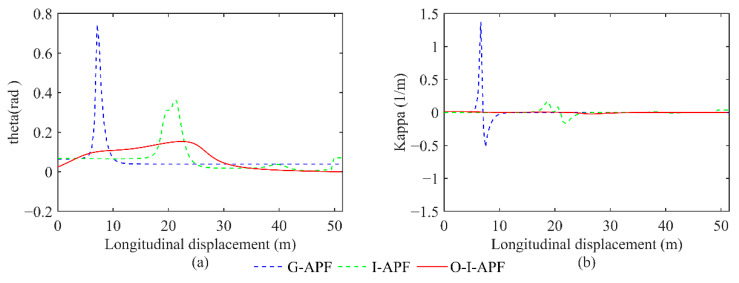
Comparison between the heading angle and curvature calculated using different algorithms. (**a**) Changes in the front wheel angle. (**b**) Changes in route curvature.

Figure 16 displays a comparison between the heading angle and curvature for the three different methods.

Figure 16 illustrates that the O-I-APF method exhibits a smoother change in the path turning angle and curvature on the planned route. Additionally, the method effectively controls the maximum curvature within 0.02, leading to improved outcomes.

### 4.2. Simulation Analysis of Path Tracking Control

To validate the practicality of the optimized path outlined in the previous section, we used the trajectory tracking controller proposed in this study to conduct tracking experiments on the path generated using the O-I-APF method. The experiment was performed using the Carsim–Simulink joint simulation platform for the aforementioned three scenarios. Considering a road’s typical driving conditions, characterized by dry and well-built asphalt pavement, the road adhesion coefficient was set to 0.8. Table 6 lists the primary vehicle parameters.

The red path outlined in the above scenario was fed into the controller for tracking control. Then, we evaluated the comparative effectiveness of the general sliding SMC for tracking path against the improved SMC (I-SMC) for tracking path proposed in this study. The simulation experiments were performed at two vehicle speeds, namely, 10 m/s and 20 m/s.

(1) Scenario 1: Changing lanes (static)

**Figure 17:**
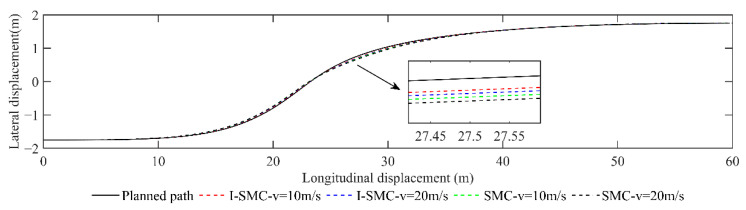
Paths tracked using different methods in scenario 1.

The lateral displacement comparison is presented in Figure 17. The figure includes various lines representing different scenarios. The solid black line represents the planned original path. The red dashed line depicts the change in vehicle displacement using the previously discussed controller at a speed of 10 m/s. Similarly, the blue dashed line represents the change in vehicle displacement using the same controller but at a speed of 20 m/s. The green dashed line illustrates the change in vehicle displacement using a general SMC controller at a speed of 10 m/s. Finally, the black dashed line showcases the change in vehicle displacement using a general SMC controller at a speed of 20 m/s.

Figure 17 demonstrates that, at velocities of 10 m/s and 20 m/s, the trajectory followed by the I-SMC controller closely aligns with the expected path, in contrast with the general SMC controller. This indicates a superior tracking accuracy of the controller proposed in this study.

**Figure 18:**
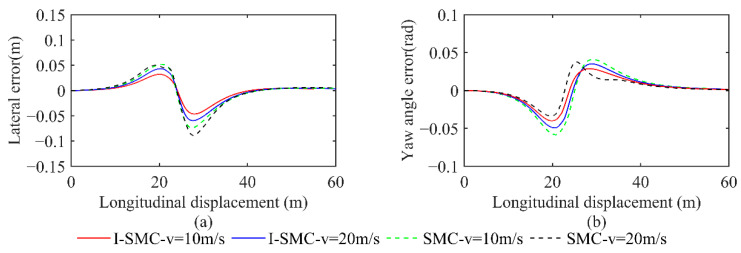
Comparison of errors in scenario 1. (**a**) Comparison of lateral errors; (**b**) Comparison of yaw angle errors.

The comparison of lateral and heading errors, acquired with various vehicle speeds and controllers, is displayed in Figure 18. The red solid line illustrates the error achieved using the controller proposed in this study at a vehicle speed of 10 m/s. Similarly, the blue solid line represents the error obtained at a vehicle speed of 20 m/s using the same controller. The green dashed line exhibits the error attained using a conventional SMC controller at a vehicle speed of 10 m/s, while the black dashed line demonstrates the error obtained at a vehicle speed of 20 m/s using the same controller.

From Figure 18, it is evident that at a low speed of 10 m/s, the general SMC results in a maximum absolute lateral error value of 0.0740 m and a maximum absolute heading error value of 0.0584 rad. In contrast, the I-SMC controller achieves a lower maximum absolute lateral error value of 0.0466, which represents a 37.03% reduction compared with the results of the general SMC controller. Similarly, the maximum absolute heading error value for the I-SMC controller is 0.0400 rad, which is 31.51% lower than the results of the general SMC controller. At a higher speed of 20 m/s, the I-SMC controller exhibits a maximum absolute lateral error value of 0.0598 m, which is 32.66% lower than the value obtained using the general SMC controller. While the absolute maximum heading angle error obtained using the I-SMC controller is 0.0493 rad, indicating a slight improvement compared with the general SMC controllers, it does show smoother variation and higher stability. Based on the experimental results, it can be concluded that the I-SMC controller effectively tracks the planned path with a smaller tracking error and better tracking accuracy.

(2) Scenario 2: Static overtaking

**Figure 19:**
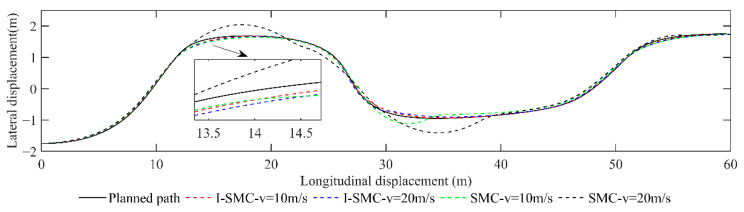
Paths tracked using different methods in scenario 2.

A comparison of lateral displacement is presented in Figure 19.

From Figure 19, it is evident that the I-SMC exhibits superior tracking performance at low speeds and demonstrates closer alignment with the expected path. In contrast, under high-speed conditions, the general SMC controller deviates significantly from the expected path, rendering the car unable to faithfully follow the planned route. Conversely, both trajectories generated using the I-SMC controller closely approximate the expected path. Consequently, the planned route is deemed feasible, and the tracking accuracy of the controller proposed in this study is relatively high.

**Figure 20:**
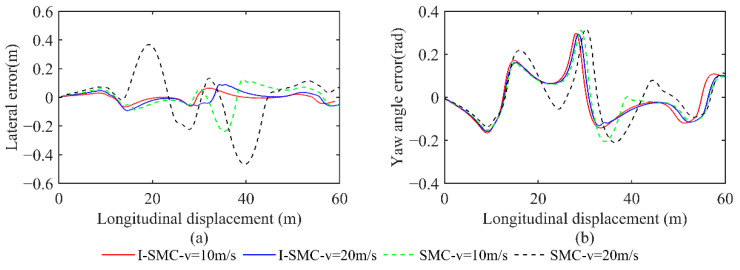
Comparison of errors in scenario 2. (**a**) Comparison of lateral errors. (**b**) Comparison of yaw angle errors.

The comparison between the lateral error and heading error collected in scenario 2 is illustrated in Figure 20.

Based on Figure 20, at a low speed of 10 m/s, the maximum absolute lateral error obtained using the general SMC is 0.2359 m, and the maximum absolute heading error is 0.3103 rad. However, the maximum absolute lateral error obtained using the I-SMC is 0.0667 m, which is 71.73% lower than the result obtained using the general SMC. The absolute value of the maximum heading error is 0.2978 rad, which is 4.03% lower than the results obtained using general SMC controllers. At a high speed of 20 m/s, the maximum absolute lateral error obtained using the improved SMC controller is 0.0923 m, which is 80.24% lower than the general SMC controller. The absolute value of the maximum heading angle error obtained using the I-SMC controller is 0.2940 rad, which is 7.08% lower than the general SMC controller. Based on the experimental results, it can be inferred that the I-SMC controller effectively tracks the planned path with reduced tracking error and improved tracking accuracy.

(3) Scenario 3: Dynamic overtaking

**Figure 21:**
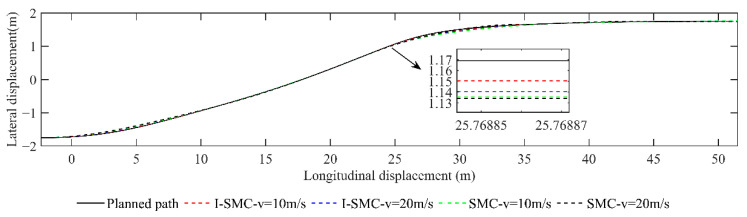
Paths tracked using different methods in scenario 2.

A comparison of lateral displacement is presented in Figure 21.

Figure 21 demonstrates that the I-SMC controller exhibits a greater degree of alignment between the planned and expected paths, regardless of the speed, in comparison with results obtained using general SMC controllers. However, the four lines closely adhere to the planned path, thereby confirming the feasibility of the path proposed in this study.

**Figure 22:**
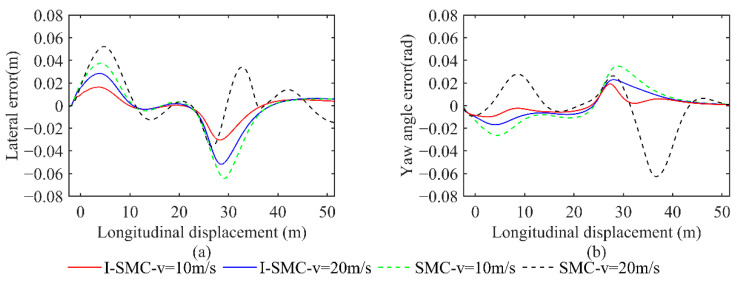
Comparison of errors in scenario 3. (**a**) Comparison of lateral errors. (**b**) Comparison of yaw angle errors.

A comparison between the lateral error and heading error collected in scenario 2 is illustrated in Figure 22.

From Figure 22, it is evident that the general SMC attains a maximum absolute lateral error of 0.0644 m and a maximum absolute heading error of 0.0350 rad when the speed is reduced to 10 m/s. Conversely, when using the I-SMC controller, the maximum absolute lateral error is 0.0304 m, exhibiting a reduction of 52.80% compared with the general SMC controller. Similarly, the absolute value of the maximum heading error is 0.0192 rad, indicating a decrease of 45.14% when compared with the general SMC controllers. At a higher speed of 20 m/s, the I-SMC controller achieves a maximum absolute lateral error of 0.0519 m, which is only 0.76% lower than that of the general SMC controller. Nonetheless, the absolute value of the maximum heading angle error obtained using the I-SMC controller is 0.0229 rad, representing a notable decrease of 63.48% in comparison with the general SMC controller. Based on the experimental results, it can be inferred that the I-SMC controller demonstrates enhanced capability in tracking the intended path with reduced tracking error and improved accuracy.

In scenario 3, the error of I-SMC is significantly lower compared with that of the SMC. The trend in error variation is similar in both low-speed and high-speed I-SMC. This study analyzed the error in low-speed I-SMC. Initially, within a longitudinal distance of 0–5 m, as the vehicle begins to turn, the lateral error increases proportionally to the turning radius. Subsequently, within a longitudinal distance of 5–10 m, the steering wheel returns to its normal position, causing the turning radius to decrease and consequently reduce the lateral error. Within a longitudinal distance of 10–22 m, the vehicle maintains a straight driving trajectory, resulting in a minimal lateral error. Within the longitudinal distance of 22–28 m, the car initiates a turn in a different direction, causing a subsequent increase in the lateral error. Between the longitudinal distance of 28 and 36 m, the vehicle enters the steering wheel alignment stage, resulting in a decrease in both the turning radius and the lateral error. Beyond a lateral distance of 36 m, the vehicle proceeds straight along the road, exhibiting no lateral error. Furthermore, the heading error varies correspondingly with the alternation in turning radius. The improved SMC method proposed in this study effectively mitigates both lateral and heading errors.

## 5. Conclusions

This study proposes an improved APF algorithm to solve a series of problems. Firstly, the range of the repulsive field is determined, allowing the range to change in real time depending on the obstacle’s speed. In addition, a road repulsion field function is incorporated. Moreover, a target point distance factor and virtual sub-target points are introduced to resolve the problems associated with unreachable targets and local minima. Furthermore, the inclusion of a velocity repulsion field enables the vehicle to adapt not only to static obstacles but also to dynamic ones. To validate the feasibility of the proposed path, an improved SMC controller is designed, which integrates both lateral and heading errors effectively. The optimized path from the planning layer is fed into the controller. The controller’s results are then compared to those obtained using the traditional SMC controller’s tracking results. Conclusively, the controller examined in this study effectively tracks three different paths in high-speed and low-speed states. Moreover, it exhibits minimal lateral and heading errors while maintaining high tracking accuracy.

In this study, we primarily explore the local path planning method for autonomous vehicles. We utilize an enhanced sliding mode controller, based on the improved artificial potential field algorithm, for trajectory tracking control. However, it is important to note that there are certain limitations associated with this approach. Firstly, our path planning methodology involves a two-degree-of-freedom ideal vehicle model, neglecting the consideration of multiple degrees of freedom. Secondly, we do not comprehensively accounted for external factors like road conditions and weather in our analysis of traffic scenarios. In future research, we aim to investigate various operating conditions and conduct more extensive studies on unexpected events such as adverse weather and other vehicle interventions, thereby making our algorithm more robust. Additionally, it should be noted that the improved artificial potential field method proposed in this study has certain computational speed drawbacks. Therefore, in the future, we intend to explore ways to simplify the calculation process. Currently, our experimentation remains limited to the simulation phase. However, we plan to validate the effectiveness of our proposed method using real vehicle experiments in subsequent research.

## Figures and Tables

**Table 1:** Comparison of Improved APF Methods.

Methods	Merits	Drawbacks
Based on the safe distance model	Driving safety	Restricted turning radius
Algorithm fusion	Real-time performance of path planning	Difficulty in setting the threshold
Improvement in the repulsive field	Driving safety, vehicle Stability	Poor adaptability to complex environments
Improvement in the gravitational field	Feasibility of path planning, vehicle Stability	Constraints of the traffic environment
Multi-condition model	Driving safety	Poor adaptability to complex environments

**Table 2:** Comparison of Improved Path Tracking Methods.

Methods	Merits	Drawbacks
Hierarchical dynamic drift controller	High tracking accuracy	Poor system stability
Discrete LQR	Simple calculations	Weak steering stability
Super twisted SMC	Good system stability	Long calculation time
Integral terminal SMC	Fast convergence speed	Poor system stability
Adaptive integral terminal SMC	Good system stability	Poor tracking accuracy

**Table 3:** Path planning results for scenario 1.

Algorithm	Length (m)	Planning Time (s)	Maximum Curvature (1/m)
G-APF	60.145	0.024	0.788
I-APF	60.286	0.011	0.201
O-I-APF	60.009	0.012	0.033

**Table 4:** Path planning results for scenario 2.

Algorithm	Length (m)	Planning Time (s)	Maximum Curvature (1/m)
G-APF	60.491	0.019	1.164
I-APF	61.635	0.009	0.797
O-I-APF	60.965	0.011	0.236

**Table 5:** Path planning results for scenario 3.

Algorithm	Length (m)	Planning Time (s)	Maximum Curvature (1/m)
G-APF	54.218	0.021	1.371
I-APF	54.114	0.016	0.167
O-I-APF	53.731	0.018	0.014

**Table 6:** Vehicle parameters.

Parameters (Units)	Value
Distance from the center of mass to the front axis a (m)	1.015
Distance from the center of mass to the rear axis b (m)	1.895
Height of the center of mass h (m)	0.54
Vehicle mass m (kg)	1270
Moment of inertia I_z_ (kg·m^2^)	1536
Effective radius of wheel r (m)	0.325
Front wheel lateral stiffness k1/(N·rad^−1^)	56,500
Rear wheel lateral stiffness k2/(N·rad^−1^)	66,500

## Data Availability

Not applicable.
